# High Burden of Prevalent and Recently Acquired HIV among Female Sex Workers and Female HIV Voluntary Testing Center Clients in Kigali, Rwanda

**DOI:** 10.1371/journal.pone.0024321

**Published:** 2011-09-19

**Authors:** Sarah L. Braunstein, Chantal M. Ingabire, Eveline Geubbels, Joseph Vyankandondera, Marie-Michèle Umulisa, Elysée Gahiro, Mireille Uwineza, Coosje J. Tuijn, Denis Nash, Janneke H. H. M. van de Wijgert

**Affiliations:** 1 Mailman School of Public Health, Columbia University, New York, New York, United States of America; 2 Projet Ubuzima, Kigali, Rwanda; 3 Academic Medical Center of the University of Amsterdam, Department of Internal Medicine, and Amsterdam Institute for Global Health and Development, Amsterdam, The Netherlands; 4 Belgian Development Agency, Kigali, Rwanda; University of Toronto, Canada

## Abstract

**Objectives:**

To estimate HIV prevalence and risk factors in population-based samples of female sex workers (FSW) and female voluntary counseling and testing (VCT) clients in Rwanda.

**Methods:**

We conducted a cross-sectional survey of 800 FSW and 1,250 female VCT clients in Rwanda, which included interviewing and testing for HIV-1/2, HSV-2 and pregnancy, and BED-CEIA and Avidity Index (AI) to identify recent infections among HIV-infected women.

**Results:**

Prevalence of HIV-1, HSV-2, and pregnancy were 24% (95% CI: 21.0–27.0), 59.8% (56.4–63.2), and 7.6% (5.8–9.5) among FSW, and 12.8% (10.9–14.6), 43.2% (40.4–46.0), and 11.4% (9.7–13.3) among VCT clients, respectively. Thirty-five percent of FSW and 25% of VCT clients had never been HIV tested. Per national guidelines, 33% of newly HIV-diagnosed FSW and 36% of VCT clients were already eligible for ART based on CD4<350 cells/µl. Condom use at last sex was higher among FSW (74%) than VCT clients (12%). In age and district of residence-adjusted models, HIV-1 seropositivity was associated with HSV-2 co-infection; recent treatment for sexually transmitted infection (STI); genital symptoms; forced sex; imprisonment; widowhood; and alcohol consumption. Eleven percent of FSW and 12% of VCT clients had recently acquired HIV-1 per BED-CEIA and AI. HSV-2 infection and recent STI treatment were associated with recent HIV infection in both groups, and being married and vaginal cleansing were associated with recent infection before last sex among VCT clients.

**Conclusions:**

This population-based survey reveals a high HIV prevalence and incidence among FSW and female VCT clients in Kigali, the scale of which is masked by the low general-population HIV prevalence in Rwanda. HIV/STI and family planning services should be strengthened.

## Introduction

Accurate and timely information on HIV prevalence and risk factors, particularly among high-risk groups, is scant in many developing countries, including Rwanda [1]. Rwanda has conducted sentinel surveillance of HIV infection since the 1980s, focusing primarily on women attending antenatal care clinics and patients receiving sexually transmitted infection (STI) treatment [1]–[4]. Several national, population-based surveys have measured HIV seroprevalence, including a 2005 Demographic and Health Survey-Plus (DHS+), which found an HIV prevalence of 3% among adults nationwide, with marked differences in prevalence by region, age and gender [5]. In the capital city of Kigali, prevalence was 6.7% among all adults, and 8.0% among adult women. More than half of HIV-1 seropositive survey participants (56% of women, 66% of men) had never been HIV tested prior to the survey [5]. However, while DHS+ provided a good general population estimate of the burden of and basic factors associated with HIV in Rwanda, infection rates and risk factors in high-risk, harder-to-reach sub-groups, such as female sex workers (FSW), may be substantially higher.

From the standpoint of HIV surveillance, FSW comprise an important group for monitoring heterosexual transmission of HIV, in early as well as late-stage epidemics, because they typically have high prevalence rates and are likely to transmit infection through frequent, often high-risk sexual contact [6]–[11]. Sex workers, as a “most-at-risk-population” (MARP), are also a key vulnerable group for targeted HIV prevention and other public health interventions [12], [13]. However, relatively few countries to date have developed long-term or community-based prevention programs for FSW, largely because of the illegality of sex work in many settings. Sex work is legal in Rwanda, and early serosurveys of FSW in the 1980s found extremely high rates of HIV infection (75% to nearly 90%) [14]. However, there has been limited systematic HIV surveillance in this group since then and, consequently, there are limited data to describe the current burden of HIV/AIDS among FSW in Rwanda, particularly from community-based samples [15].

Clients of voluntary counseling and testing (VCT) centers are another important group from the perspective of HIV surveillance and primary and secondary prevention [16]–[18]. VCT is a key component of HIV prevention and control interventions, and information about clients' risk profiles can help inform VCT-based interventions. Furthermore, clients testing because of HIV exposure, including those in serodiscordant relationships, or perceived high risk are important groups for reinforcement of prevention messages. Finally, VCT clients are easily accessible because they themselves come forward for HIV testing. Despite concerns about the representativeness of this group compared to the general population [19], data from VCT clients can nonetheless be an important source of information on HIV prevalence [20].

We conducted a cross-sectional survey to screen 800 FSW and 1,250 female VCT clients for an HIV incidence study at Projet Ubuzima, a non-governmental organization for medical research based in Kigali, Rwanda. The primary aim of the study was apply and validate tests for early HIV infection. However, an important secondary aim of the survey was to describe the epidemiology of HIV in these sentinel groups. This paper presents HIV-1/2 prevalence in the two groups, including the proportion of HIV-positive individuals with recent versus long-term infection (based on testing by the BED-CEIA and Avidity Index assays); prevalence of herpes simplex virus type 2 (HSV-2) and pregnancy; demographic and behavioral characteristics; and a comparison of factors associated with prevalent and recently acquired HIV infection.

## Materials and Methods

### Ethics statement

All study participants provided written informed consent prior to study participation. The study was approved by the National Ethics Committee and the National AIDS Control Commission (CNLS) in Rwanda, and the Columbia University Medical Center Institutional Review Board in the United States.

### Recruitment, screening and enrollment

#### Female sex workers

FSW were recruited via community meetings in 3 Kigali districts, after approval from local authorities. Meetings were led by Projet Ubuzima outreach staff with assistance from prominent community members with extensive social networks, some of whom were working as sex workers themselves (“community mobilizers”). At recruitment sessions, women who passed a short pre-screening questionnaire were invited to the study clinic for a full eligibility assessment. Eligible women were: ≥18 years; at high risk for sexual exposure to HIV, defined as having exchanged sex for money at least once in the last month; of either unknown HIV serostatus or had a last test that was negative; and willing and able to provide written informed consent. The first 800 eligible women who participated in the survey comprised the final sample.

#### Female VCT clients

Female VCT clients were recruited from 11 public HIV testing centers around Kigali city. All female clients presenting at the center on the designated recruitment day were screened for study eligibility (based on age ≥18 years, and willingness to provide written informed consent) by a study nurse/counselor. Each VCT site had its own recruitment target based primarily on clientele volume, and recruitment was conducted successively across sites until the total sample size of 1,250 was reached. Sample sizes for both groups were based on estimates of HIV prevalence, and the projected recruitment targets during the study period.

### Study procedures

All study procedures for FSW took place at the Projet Ubuzima study clinic, and sample collection and interviewing for VCT clients was done onsite at the VCT centers. All participants provided written informed consent and eligibility was confirmed. Per the Rwandan National Ethics Committee, individuals aged 18–20 in both groups were required to obtain parental/guardian consent for study participation. However, as VCT clients would have had to return the next day with a parent/guardian, we did not recruit VCT clients aged 18–20. Blood specimens were collected for HIV, pregnancy and HSV-2 testing. A face-to-face interview was conducted to collect information on demographics, medical and reproductive history, including HIV testing history, partnership status and sexual behavior. Women were given HIV and pregnancy test results and an appointment card to return to receive HSV-2 results and CD4 results (if HIV positive).

All HIV testing was accompanied by pre- and post-test counseling and followed a slightly modified national testing protocol: a First Response Rapid Test (Premier Medical Corporation, India); if positive, confirmation by Uni-Gold Rapid Test (Trinity Biotech Plc, Ireland); and Capillus HIV-1/HIV-2 Rapid Test (Trinity Biotech Plc, Ireland) as a tie-breaker if needed. HIV rapid test-positive specimens were confirmed by Murex HIV Ag/Ab Combination ELISA (Abbott Laboratories, Germany); tested by CD4 cytometry; and tested by the BED-CEIA (BED) assay (Calypte Biomedical Corporation, USA) [21] and Avidity Index (AI) method (using the AxSYM ELISA platform, Abbott Laboratories, USA) [22] to distinguish recent HIV infection (<6 months) from chronic HIV infection. Blood specimens were also tested by the Fortress (Fortress Diagnostics, UK) or Orea (Global Diagnostics, UK) hCG serum pregnancy test, and HSV-2 HerpeSelect 2 ELISA (Focus Technologies, USA). All testing was done at Projet Ubuzima or the National Reference Laboratory in Kigali. HIV test results were not available for 3 VCT clients; CD4 results were not available for 3 FSW and 31 VCT clients; and BED/AI results were not available for 3 FSW and 5 VCT clients.

Women who tested HIV positive were referred to a local health center for care and evaluation for treatment eligibility, as well as psychosocial services. Pregnant women were referred for prenatal care, and HSV-2 seropositive women were counseled to seek care in the event of a herpes outbreak. FSW received 4,000 Rwandan Francs (approximately 7 USD) as compensation for travel to and time spent at the study clinic, and VCT participants received 500 Rwandan Francs (approximately 1 USD) to cover the standard fee for HIV VCT in Rwanda.

### Statistical analysis and outcome definitions

Descriptive statistics were used to summarize demographic and behavioral characteristics of study participants. Categorical variables are expressed as percentages, and continuous data as medians and inter-quartile ranges (IQR). HIV, HSV-2 and pregnancy prevalence were measured as the proportion of individuals with a positive test result during the survey, and are presented with 95% confidence intervals (CI) for the binomial proportion. The Cochran-Armitage trend test was used to calculate p-values for HIV-1 and pregnancy status by age group, and the Chi-square test was used to calculate p-values for HIV-1 status by district of residence (Gasabo, Kicukiro, Nyarugenge combined versus outside Kigali).

Prevalent HIV-1/2 infection was identified by positive HIV rapid test, with ELISA confirmation. Demographic and behavioral covariates were analyzed individually in separate logistic regression models, each of which was adjusted for age and district of residence, to assess their independent association with prevalent HIV infection, with p-values from the Wilcoxon-Mann-Whitney test for continuous variables and the Chi-square and Fisher's exact tests for categorical variables. Parsimonious models were built for prevalent infection (versus fully-derived multivariable models) to enable comparison with results from models for recent infection, for which there were small numbers of outcomes.

Correlates of recent HIV infection (based on BED and AI assay results) were assessed individually in separate logistic regression models that adjusted for age and residence, comparing individuals with recent HIV infection (RI) to HIV-negative individuals. In order to maximize specificity, we took a conservative approach for the risk factor analysis of RI, such that only individuals classified concordantly as RI on the BED and AI, and with CD4≥200 cells/µl, were considered true RI cases. Samples with discordant assay classifications, those classified concordantly as RI but with CD4<200, and those classified concordantly as RI but without CD4 results, were excluded. Detailed information regarding the validity of the BED and AI assays has been reported elsewhere [23]. All tests were two-sided. Analyses were performed in SAS, version 9.1.3 (SAS Institute, Inc., Cary, NC).

## Results

### Screening and enrollment

Prior to the survey, 1,238 FSW were pre-screened by study staff for potential eligibility; 116 women were determined to be ineligible because of age <18 years (n = 14), known HIV-positive status (n = 85), or low HIV risk (n = 17). The first 800 eligible women who participated in the survey comprised the final FSW sample. Data on reasons for ineligibility among the VCT client sample were not collected.

### HIV, HSV-2 and pregnancy prevalence

HIV-1 prevalence was 24.0% (95% CI 21.0–27.0) among FSW, and 12.8% (10.9–14.6) among VCT clients (Table 1). In both groups, HIV-1 prevalence significantly increased with age, varied by district of residence, and among VCT clients, also varied by testing site. Prevalence of HIV-2 was 0.75% (95% CI: 0.15–1.4) among FSW, and 1.8% (1.1–2.8) among VCT clients. All HIV-2-positive women in both groups were also HIV-1 positive.

**Table 1:** Prevalence of HIV, HSV-2 and pregnancy among female sex workers and female VCT clients in Kigali, Rwanda.

	Female sex workers (N = 800)		Female VCT clients (N = 1,250)	
	% (95% CI)	*P value*	% (95% CI)	*P value*
**HIV-1**	24.0 (21.0, 27.0)	NA	12.8 (10.9, 14.6)	NA
**HIV-1 by age (in years):**				
18–20	16.1 (7.0, 25.3)		0	
21–24	17.4 (12.8, 22.0)	<0.001	8.8 (6.5, 11.5)	<0.001
25–29	23.3 (18.1, 28.5)		11.1 (7.9, 15.0)	
30–34	33.6 (25.1, 42.1)		21.4 (15.2, 28.8)	
≥35	33.7 (24.7, 42.6)		18.2 (13.5, 23.8)	
**HIV-1 by district of residence:**				
Gasabo	24.1 (19.2, 29.1)		9.7 (7.3, 12.4)	
Kicukiro	33.2 (27.1, 39.3)	<0.0001	16.2 (12.7, 20.3)	0.02
Nyarugenge	16.1 (11.8, 20.5)		17.5 (12.8, 23.1)	
Other/outside Kigali	NA		4.1 (0.9, 11.5)	
**HIV-1 by VCT site:**				
Gitega Health Center			15.0 (8.9, 23.5)	
ARBEF			12.0 (7.6, 17.8)	
Muhima Hospital			16.0 (8.6, 26.3)	
Kimironko Health Center			21.0 (13.5, 30.3)	
Kacyiru Health Center	NA		14.7 (7.6, 24.7)	<0.001
Kinyinya Health Center			10.4 (5.7, 17.1)	
Kanombe Hospital			20.0 (11.7, 30.8)	
Gahanga Health Center			5.3 (1.5, 13.1)	
Kicukiro Health Center			15.6 (10.7, 21.8)	
L'Association des Guides			3.5 (1.3, 7.4)	
Gikondo Health Center			13.7 (7.5, 22.3)	
**HIV-2**	0.75 (0.15, 1.4)	NA	1.8 (1.1, 2.8)	NA
**HSV-2**	59.8 (56.4, 63.2)	NA	43.2 (40.4, 46.0)	NA
Among HIV positive, % HSV-2 infected	86.8 (82.0, 91.6)		84.3 (77.7, 89.6)	
**Pregnancy by age (in years):**	7.6 (5.8, 9.5)		11.4 (9.7, 13.3)	
18–20	6.5 (0.3, 12.6)		0	
21–24	10.8 (7.0, 14.6)		12.5 (9.8, 15.7)	
25–29	6.7 (3.6, 9.8)	0.10	11.7 (8.5, 15.7)	0.07
30–34	5.0 (1.1, 9.0)		13.0 (8.1, 19.3)	
≥35	5.6 (1.3, 10.0)		7.2 (4.3, 11.3)	
Among currently pregnant women, % HIV+	16.4 (7.1, 25.7)		12.0 (7.7, 19.3)	

Seroprevalence of HSV-2 was 59.8% (95% CI: 56.4–63.2) among FSW, and 43.2% (40.4–46.0) among VCT clients. Nearly 87% of HIV-1-infected FSW and 84% of HIV-1-infected VCT clients were co-infected with HSV-2 (hereafter, all references to “HIV” refer to HIV-1 infection only, unless otherwise indicated). Among FSW, there was a positive association between prevalent HSV-2 and number of years working as a sex worker (age- and district-adjusted odds ratio [AOR] comparing 6–10 vs. 2 or fewer years: 1.9 (95% CI: 1.1–3.5)).

Pregnancy prevalence among FSW was 7.6% (95% CI: 5.8–9.5), with the highest prevalence among women aged 21–24 (10.8%, 95% CI: 7.0–14.6) and lowest among those aged 30–34 (5.0%, 95% CI: 1.1–9.0). Pregnancy prevalence was higher among VCT clients (overall 11.4% (9.7–13.3)). Sixteen percent of pregnant FSW and 12% of currently pregnant VCT clients tested HIV positive in the survey.

Among HIV-positive women with CD4 results available, 42% of FSW and 38% of VCT clients had a CD4 count ≥500 cells/µl, while 12% of FSW and 15% of VCT clients had ≤200 CD4 cells/µl (Table 2). Fifteen percent of the total FSW sample and 10% of the VCT sample reported at least one recent AIDS symptom, including recent unexpected weight loss, chronic diarrhea, chronic weakness, fever, cough, night sweats, or oral candidiasis (Table 2). Among HIV-positive women in both groups, the proportion of women reporting such symptoms was inversely related to CD4 count. In both groups, HSV-2 co-infection rates decreased with higher CD4 counts.

**Table 2:** CD4 count distribution among HIV-positive female sex workers and female VCT clients in Kigali, Rwanda.

CD4 range (cells/µl)	All women % (N)	% Reporting AIDS symptom in past 6 months	% Pregnant	% HSV-2 seropositive
**Female sex workers (N = 189)**				
<100	4.2 (8)	25.0 (2)	0 (0)	100 (8)
100–199	7.4 (14)	21.4 (3)	0 (0)	93 (13)
200–349	21.1 (40)	22.5 (9)	2.5 (1)	97.5 (39)
350–499	25.3 (48)	20.8 (10)	8.3 (4)	83.3 (40)
≥500	41.8 (79)	17.7 (14)	6.3 (5)	81.3 (65)
**Female VCT clients (N = 128)**				
<100	3.9 (5)	20.0 (1)	0 (0)	100 (5)
100–199	10.9 (14)	28.6 (4)	21.4 (3)	85.7 (12)
200–349	21.1 (27)	18.5 (5)	7.4 (2)	100 (27)
350–499	26.6 (34)	14.7 (5)	11.8 (4)	82.4 (28)
≥500	37.5 (48)	14.6 (7)	10.4 (5)	81.3 (39)

### Sociodemographics and contraceptive use

The median age among both FSW and VCT clients was 25 years, with VCT clients slightly older than FSW (Table 3). Overall, 92% of FSW and 45% of VCT clients reported currently using a modern or traditional (e.g., periodic timed abstinence) contraceptive method, with the majority of users (97% FSW, 68% VCT clients) reporting modern methods. In both groups, the most common methods were male condoms and injectable hormonal methods (80% and 13%, respectively, for FSW; 15% and 10%, respectively, for VCT clients). FSW had had a median of 2 pregnancies during their lifetimes, with only 7% reporting no pregnancies; 43% were currently breastfeeding. VCT clients had had a median of 1 pregnancy during their lifetimes, with 32% reporting no pregnancies; 24% of VCT clients were currently breastfeeding.

**Table 3:** Demographic and behavioral characteristics of female sex workers and female VCT clients, Kigali, Rwanda.

Demographic characteristics	Female sex workers (N = 800)	Female VCT clients (N = 1,250)
Median age, in years (range)	25.0 (18–49)	25.0 (20–68)
*Age groups*, N(%):		
18–24	360 (45)	527 (42)
25–29	230 (29)	333 (27)
30–34	114 (14)	154 (12)
≥35	96 (12)	236 (19)
Education level, N(%)		
No formal schooling	166 (21)	194 (16)
Some primary school	326 (41)	347 (28)
Completed primary school	207 (26)	386 (32)
Secondary school (partial or completed)	94 (12)	277 (22)
Post-secondary	5 (<1)	26 (2)
Currently using family planning method, N(%)	732 (92)	555 (45)
Median no. lifetime pregnancies (IQR)	2 (1–3)	1 (0–3)
Currently breastfeeding, N(%)	344 (43)	279 (24)
Marital status, N(%)		
Married (legal or common-law marriage)	4 (1)	452 (36)
Divorced/separated	117 (15)	145 (12)
Widowed	93 (12)	96 (8)
Never married	584 (73)	557 (45)
Currently have steady partner	275 (34)	220 (18)
Vaginal sex in last month, N(%)	797 (99.6)	677 (54)
At last sex:		
Received money or gift from partner	744 (93)	23 (2)
Partner used a condom	593 (74)	150 (12)
Median no. vaginal sex acts in past month (IQR)	40 (20–64)	1(0–8)
Median no. different sex partners in last 3 months (IQR)	96 (56–180)	1 (0–1)
Condom use with casual/paying partner, N(%)	(N = 800)	(N = 99)
Never	11 (1)	50 (51)
Rarely	128 (16)	10 (10)
Half of the time	91 (12)	3 (3)
Most of the time, but not always	403 (50)	16 (16)
Always	167 (21)	20 (20)
Condom use with steady partner, N(%)	(N = 275)	(N = 230)
Never	171 (63)	123 (54)
Rarely	36 (14)	35 (15)
Half of the time	7 (3)	10 (4)
Most of the time, but not always	11 (4)	21 (9)
Always	45 (17)	41 (18)
Condom use with husband, N(%)	(N = 4)	(N = 452)
Never	2 (50)	358 (78)
Rarely	1 (25)	38 (8)
Half of the time	0	10 (2)
Most of the time, but not always	0	10 (2)
Always	1 (25)	40 (9)
Median no. clients per week in last month (IQR)	10 (5–16)	NA
Median years working as sex worker (IQR)	3 (2–5)	NA
Have sex partner known to be HIV positive, N(%)	72 (9)	150 (12)
Ever had forced sex, N(%)	202 (25)	254 (20)
Median no. lifetime HIV tests (IQR)	1 (0–2)	2 (1–3)
Never tested, N(%)	278 (35)	306 (25)
Once	278 (35)	314 (25)
Twice	115 (14)	264 (21)
3–5 times	106 (13)	300 (24)
≥6 times	21 (3)	61 5)
≥1 Genital symptom in past month, N(%)	173 (22)	231 (19)
Sought treatment for STI symptom in last 3 months, N(%)	91 (11)	113 (9)
Drink alcohol regularly, N(%)	436 (55)	139 (11)
Ever imprisoned, N(%)	317 (40)	78 (6)

### Marital status, partnerships and sexual behavior

Over one-third (36%) of VCT clients were currently married, compared with <1% of FSW. HIV prevalence among married VCT clients was 14.7%, versus 11.9% among unmarried VCT clients. Among married VCT clients, 75% had a husband at least 1 year older than they, and 26% of these women had a husband ≥10 years older. Twelve percent of FSW and 8% of VCT clients had been widowed. Among widows, the most common reasons were war (42% of FSW widows, 36% of VCT widows); AIDS or other illnesses (26% FSW widows, 40% VCT widows); and motor vehicle and other accidents (19% FSW widows, 12% VCT widows).

In addition to paying clients, 23% of FSW had casual (non-paying) sex partners, and more than one-third (34%) also had a steady partner (defined as a regular sex partner with whom the woman has sex more often than with other sex partner(s), but does not live with and is not married to). Most FSW reported finding clients at bars/nightclubs (60%) or on the street (47%), and that sex typically takes place in public places (42%), at clients' homes (40%), or the woman's home (34%). Compared with FSW, fewer VCT clients had casual (4%) or steady partners (18%).

Seventy-four percent of FSW reported their last sex partner used a condom, versus only 12% of VCT clients. Condom use with steady partners and husbands was low (50–80% reported never using condoms) in both groups. Nine and 12% of FSW and VCT clients, respectively, reported having a current sex partner they knew to be HIV positive.

### HIV testing history

Per the study protocol which included longitudinal follow-up for FSW only, FSW who had a negative HIV test in the past were eligible for the study, but only if the most recent test result was negative; there was no exclusion criterion related to HIV serostatus for VCT clients who were only screened for participation in the cross-sectional survey. VCT clients reported a more frequent and recent testing history: 22% had been tested in the past 6 months (vs. 11% of FSW), and 88% of those with a history of HIV testing had had their most recent test within the past 2 years (vs. 60% of FSW). In both groups, HIV prevalence rates were higher among women who had never had an HIV test than in those who had tested before (35% vs. 18% overall for FSW (p<0.01), and 18% vs. 11% overall for VCT clients (p<0.01)). Furthermore, HIV prevalence among FSW who reported a negative result at their last HIV test was 17.5% (95% CI: 14.2, 20.9), and prevalence was 25.7% (95% CI: 22.6, 28.9) among FSW who reported not knowing their HIV status at the time of the survey.

### Factors associated with prevalent and recent HIV infection among FSW

Factors with the strongest positive associations with prevalent HIV among FSW after adjusting for age and district of residence were: HSV-2 infection (AOR 7.9, 95% CI: 4.5–13.8); history of forced sex (AOR 2.2, 95% CI: 1.5–3.2); and having sought STI treatment in the previous 3 months (AOR 2.2, 95% CI: 1.3–3.5) (Table 4). Being widowed, history of imprisonment, alcohol consumption, having a recent AIDS symptom or genital symptom (including genital itching, burning, rash, pain; abnormal vaginal discharge, odor, bleeding; pain or difficulty urinating; genital ulcers, sores, blisters; pain during sex; acute lower abdominal pain; other), and vaginal cleansing before last sex were also significantly positively associated with prevalent HIV infection. Current breastfeeding and more frequent and recent HIV testing were associated with decreased odds of prevalent infection.

**Table 4:** Factors associated with prevalent and recent HIV infection among female sex workers, Kigali, Rwanda.

Risk factor	No. HIV+ (Total N = 192)	Adjusted Odds Ratio (95% CI)	*P value*	No. Recent Infection (N = 21)	Adjusted Odds Ratio (95% CI)	*P value*
Current age (in years):						
18–24	24	– REF –		11	–REF–	
25–29	31	1.4 (1.0, 2.2)	0.08	6	0.9 (0.3, 2.5)	0.84
30–34	21	2.5 (1.6, 4.0)	<0.001	2	0.7 (0.2, 3.4)	0.69
≥35	19	2.4 (1.5, 3.9)	<0.001	2	0.9 (0.2, 3.9)	0.83
District of residence:						
Nyarugenge	45	– REF –		5	– REF –	
Gasabo	71	1.6 (1.1, 2.5)	0.02	10	2.1 (0.7, 6.2)	0.19
Kicukiro	76	2.5 (1.7, 3.8)	<0.001	6	1.8 (0.5, 6.0)	0.34
Currently breastfeeding	40	0.3 (0.2, 0.5)	**<0.001**	10	0.9 (0.4, 2.3)	0.86
Marital status						
Never married	130	1.0 (0.6, 1.5)	0.85	16	0.9 (0.3, 2.8)	0.89
Divorced	24	0.7 (0.4, 1.1)	0.13	3	1.0 (0.3, 3.7)	0.95
Widowed	37	1.8 (1.1, 3.1)	**0.03**	2	1.3 (0.3, 6.9)	0.75
Used condom at last sex	138	0.9 (0.6, 1.3)	0.49	12	0.4 (0.2, 1.0)	0.06
Cleansed vagina before last sex	100	1.5 (1.1, 2.1)	**0.02**	12	1.9 (0.8, 4.6)	0.17
Years working as sex worker:						
≤1	29	– REF –		10	– REF –	0.11
2–3	60	0.8 (0.5, 1.4)	0.51	4	0.4 (0.1, 1.4)	0.13
4–5	35	0.9 (0.5, 1.6)	0.67	4	0.7 (0.2, 2.7)	0.61
≥6	54	1.0 (0.6, 1.9)	0.90	3	0.5 (0.1, 2.2)	0.37
History of forced sex	72	2.2 (1.5, 3.2)	**<0.001**	8	2.3 (0.9, 5.8)	0.07
No. vaginal sex acts in past month:						
<20	31	0.7 (0.4, 1.2)	0.16	4	2.3 (0.4, 12.7)	0.34
20–39	57	0.9 (0.6, 1.4)	0.60	10	3.6 (0.8, 16.9)	0.10
40–59	42	0.9 (0.5, 1.5)	0.65	4	2.0 (0.4, 10.9)	0.44
60–89	45	– REF –		2	–REF–	
≥90	17	0.8 (0.4, 1.5)	0.45	1	1.0 (0.1, 11.2)	1.0
No. clients per week in last month:						
<5	31	0.7 (0.4, 1.2)	0.14	5	2.1 (0.4, 11.2)	0.37
5–9	59	1.1 (0.7, 1.8)	0.75	9	3.2 (0.7, 15.1)	0.14
10–15	52	0.9 (0.6, 1.5)	0.69	4	1.3 (0.2, 7.2)	0.77
16–25	39	– REF –		2	–REF–	
>25	10	0.7 (0.3, 1.6)	0.38	1	1.2 (0.1, 13.3)	0.90
Have an HIV+ sex partner	11	0.5 (0.3, 1.0)	**0.05**	1	0.4 (0.1, 3.3)	
No. lifetime HIV tests:						
None	97	– REF –		7	–REF–	
1	64	0.6 (0.4, 0.9)	**0.02**	5	0.6 (0.2, 1.9)	0.39
≥2	31	0.3 (0.2 0.5)	**<0.01**	9	1.1 (0.4, 3.1)	0.82
HIV tested in past 6 months	4	0.2 (0.1, 0.5)	**<0.001**	1	0.3 (0.1, 2.4)	0.27
Had ≥1 genital symptom in last month	50	1.5 (1.0, 2.2)	**0.05**	7	1.9 (0.7, 4.8)	0.20
Sought STI treatment in last 3 months	34	2.2 (1.3, 3.5)	**<0.01**	6	3.8 (1.4, 10.3)	**<0.01**
Regular alcohol consumption	120	1.5 (1.0, 2.1)	**0.03**	9	0.6 (0.3, 1.5)	0.31
History of imprisonment	89	1.7 (1.2, 2.4)	**0.01**	6	0.6 (0.2, 1.5)	0.25
HSV-2 seropositive	167	7.9 (4.5, 13.8)	**<0.001**	20	17.0 (2.3, 128.6)	**<0.01**
Currently pregnant	10	0.6 (0.3, 1.1)	0.11	2	1.0 (0.2, 4.4)	0.97

After excluding women with discordant BED and AI results, and those with CD4<200 cells/µl and therefore probable long-term infection, 11% (21/190) of HIV-positive FSW were classified by both the BED-CEIA and AI as having recently acquired HIV infection (Table 4). After adjusting for age and district of residence, the odds of recent HIV infection were higher among HSV-2 seropositive women (AOR 17.0, 95% CI: 2.3–128.6) and among women who recently sought STI treatment (AOR 3.8, 95% CI: 1.4–10.3).

Never having been married, being divorced/separated, condom use at last sex, frequency of sex, number of clients, and current pregnancy were not associated with either prevalent or recent HIV infection among FSW.

### Factors associated with prevalent and recent HIV infection among female VCT clients

After adjusting for age and district of residence, having a known HIV-positive sex partner was the variable that had the largest association with prevalent HIV infection among VCT clients (AOR 3.4, 95% CI: 1.6–7.6) (Table 5). In this group, divorced women had an increased risk of prevalent HIV (AOR 1.8, 95% CI: 1.1–2.8), while never-married women had a lower risk (AOR 0.6, 95% CI: 0.4–0.9). Similar to FSW, the risk of prevalent infection among VCT clients decreased with increasing numbers of HIV tests (AOR 0.3 for ≥4 vs. 0 lifetime tests, 95% CI: 0.2–0.6). Increased risk for prevalent HIV was also associated with HSV-2 seropositivity, history of imprisonment, recent AIDS symptom or genital symptom, recent STI treatment, and sexual activity (1–5 partners in the past month vs. none).

**Table 5:** Factors associated with prevalent and recent HIV infection among female VCT clients, Kigali, Rwanda.

Risk factor	No. HIV+ (Total N = 161)	Adjusted Odds Ratio (95% CI)	*P value*	No. Recent Infection (N = 20)	Adjusted Odds Ratio (95% CI)	*P value*
Current age (in years):						
18–24	48	– REF –		7	– REF –	
25–29	37	1.2 (0.8, 2.0)	0.35	3	0.7 (0.2, 2.7)	0.59
30–34	33	2.7 (1.7, 4.4)	**<0.001**	2	1.1 (0.2, 5.5)	0.89
≥35	43	2.2 (1.4, 3.5)	**<0.001**	8	2.8 (1.0, 7.9)	**0.05**
District of residence:						
Nyarugenge	55	– REF –		5	– REF –	
Gasabo	62	0.5 (0.3, 0.8)	**<0.01**	8	0.3 (0.1, 0.9)	**0.03**
Kicukiro	41	0.9 (0.6, 1.4)	0.58	7	0.7 (0.2, 1.9)	0.44
Outside Kigali city	3	0.2 (0.1, 0.7)	**<0.01**	0	NA	
Currently breastfeeding	31	0.8 (0.5, 1.3)	0.34	7	1.9 (0.7, 5.0)	0.18
Marital status						
Currently married	66	1.0 (0.7, 1.5)	0.84	12	2.7 (1.1, 6.9)	**0.04**
Never married	44	0.6 (0.4, 0.9)	**<0.01**	2	0.1 (0.03, 0.6)	**<0.01**
Divorced	32	1.8 (1.1, 2.8)	**0.01**	4	1.8 (0.6, 5.9)	0.31
Widowed	19	1.2 (0.7, 2.2)	0.56	2	0.6 (0.1, 2.8)	0.48
Used condom at last sex	33	2.1 (1.4, 3.3)	**<0.001**	2	0.9 (0.2, 3.9)	0.86
Cleansed vagina before last sex	10	1.0 (0.5, 2.0)	1.0	4	3.4 (1.0, 11.5)	**0.05**
History of forced sex	32	0.9 (0.6, 1.4)	0.78	2	0.4 (0.1, 1.8)	0.24
No. vaginal sex acts in past month:						
0	66	– REF –		6	– REF –	
1–5	45	1.6 (1.0, 2.4)	**0.03**	6	2.3 (0.7, 7.3)	0.16
6–15	36	1.3 (0.9, 2.1)	0.20	6	2.9 (0.9, 9.3)	0.08
≥16	14	0.8 (0.4, 1.5)	0.46	2	1.4 (0.3, 7.0)	0.70
Have an HIV+ sex partner	11	3.4 (1.6, 7.6)	**<0.01**	1	2.7 (0.3, 23.3)	0.36
No. lifetime HIV tests:						
None	56	– REF –		3	– REF –	
1	47	0.7 (0.5, 1.1)	0.17	4	1.2 (0.3, 5.3)	0.84
2	25	0.4 (0.2, 0.7)	**<0.001**	2	0.6 (0.1, 3.8)	0.61
3	14	0.3 (0.2, 0.6)	**<0.001**	2	1.0 (0.2, 6.3)	0.98
≥4	17	0.3 (0.2, 0.6)	**<0.001**	9	3.9 (1.0, 15.2)	**0.05**
HIV tested in past 6 months	19	0.5 (0.3, 0.7)	**<0.01**	3	0.6 (0.2, 2.1)	0.43
Had ≥1 genital symptom in last month	53	2.5 (1.7, 3.6)	**<0.001**	5	1.6 (0.6, 4.6)	0.36
Sought STI treatment in last 3 months	27	2.1 (1.3, 3.5)	**<0.01**	4	2.4 (0.7, 7.4)	0.15
Regular alcohol consumption	20	1.0 (0.6, 1.8)	0.88	1	0.4 (0.1, 2.9)	0.34
History of imprisonment	21	2.7 (1.6, 4.7)	**<0.001**	2	1.6 (0.4, 7.1)	0.56
HSV-2 seropositive	135	3.1 (2.4, 4.0)	**<0.001**	18	3.8 (1.7, 8.2)	**<0.001**
Currently pregnant	19	1.1 (0.7, 1.9)	0.68	0	NA	NA

After excluding 7 participants from the recent infection group (1 with CD4<200 cells/µl and 6 missing CD4 results), 20 women or 12.4% of HIV-positive VCT clients were classified as having a recent infection by both the BED-CEIA and AI assays (Table 5). After adjusting for age and district of residence, the odds of recent HIV infection were higher among currently married women (AOR 2.7, 95% CI: 1.1–6.9), women who cleansed the vagina before their last sexual encounter (AOR3.4, 95% CI: 1.0–11.5), had ≥4 HIV tests in their lifetime vs. none (AOR 3.9, 95% CI: 1.0–15.2), and were HSV-2 seropositive (AOR 3.8, 95% CI: 1.7–8.2). Current breastfeeding, widowhood, history of forced sex, regular alcohol consumption, and current pregnancy were not associated with either prevalent or recent HIV infection among VCT clients.

## Discussion

In our community-based sample of FSW in Kigali, overall HIV prevalence was 24%, and HIV seropositivity rates increased with age. All positive HIV tests represented first-time diagnoses for these women, given the exclusion criterion of having a known positive HIV status. Indeed, prevalence was even higher among FSW who reported not knowing their HIV status at the time of the survey (26%). Overall prevalence among FSW in our study is 3 times the 2005 DHS+ estimate of 8.0% HIV prevalence among women in Kigali [5], although it falls within the general range reported by other studies among sex workers in the sub-Saharan region [11], [24]–[30]. It is likely, however, that HIV prevalence among FSW was underestimated in our study because we excluded known HIV-positives. Indeed, a recent nationally-representative survey of Rwandan FSW ages 15–49 found an overall HIV prevalence of 59% (*unpublished data, TRAC Plus*). Nonetheless, our survey found evidence of recent HIV acquisition in this group, with antibody-based incidence assay testing indicating that 11% of HIV-positive participants were infected within the past 6 months. The high HIV burden, including ongoing transmission, justifies scale-up of prevention programs and systematic surveillance in FSW in Rwanda. HIV-related interventions and programs should be tailored to sex workers' needs, for example community-based health education, improved access to low-cost or free condoms, more widely available clinical services that integrate HIV testing, care and treatment with other routine care, targeted HIV testing campaigns that enable earlier detection of HIV among FSW, and support programs for FSW living with HIV/AIDS. Although we did not recruit FSW younger than 18 years, HIV prevalence in the 18–20 year old age group was already quite high, suggesting the need for education and prevention efforts targeting younger women. Our study demonstrates that, despite their potential for marginalization, FSW in Kigali can be enumerated and reached by public health programs.

HIV prevalence was also high among the urban female VCT clients we surveyed, at nearly 13%. This is more than 4 times the 2005 DHS+ national prevalence estimate of 3%, and almost twice the DHS' 8% prevalence among women in Kigali. Meaningful comparison between our VCT sample and testers from the Rwanda DHS+ is difficult, principally because only 10% of women in the DHS+ sample were living in Kigali, and urban/rural differences persist in Rwanda [1]. This is underscored by compositional differences on factors related to HIV infection between the samples (e.g., 48% of DHS+ women were married vs. 36% of our participants). However, HIV prevalence (13%) and the proportion of women with a recent infection (12%) in our sample were comparable to other studies among VCT clients in other East African countries [19], [31].

Based on parsimonious models, several factors were positively associated with prevalent HIV infection in these populations, including: older age, HSV-2 infection, recent STI treatment, history of forced sex and imprisonment, AIDS-like symptoms, widowhood, and alcohol consumption. Two of these factors—HSV-2 infection and recent STI treatment—were also positively associated with recent HIV infection in both populations. Among VCT clients, risk for recent HIV infection was increased among currently married women, and lower among those who had never been married. While these associations have been documented by others [8], [32]–[34], they suggest several possible avenues for intervention in the Rwandan context, including, for example, further investigation into the interaction between alcohol use and sex, expanded prevention and care services for widows, and perhaps social and legal advocacy related to gender-based violence. The association in this study between current marriage and recent HIV infection is difficult to interpret without more detailed information, for example on the duration and stability of marriages and risk behavior of partners. Finally, the association between HIV seropositivity and recent STI treatment suggests missed opportunities to identify HIV infections at health centers providing STI services, and therefore a need for better integration of HIV and STI screening and treatment, especially for at-risk groups such FSW and their partners.

We found a negative association between more frequent and recent HIV testing and prevalent HIV in both groups. This could reflect greater likelihood of testing among women with a high perceived but low actual risk for HIV. Alternatively, testing itself may confer some protective benefit against infection, for example promoting safer sexual behavior to maintain a negative serostatus. However, the cross-sectional study design makes it difficult to identify specific dynamics related to motivation for HIV testing, as well as the timing of testing relative to risk behavior. The negative association between testing history and prevalent HIV risk among FSW could also reflect selection bias from exclusion of FSW with known positive HIV serostatus from the study. Furthermore, the positive association we found between recent HIV infection and frequent HIV testing among VCT clients may reflect a detection bias, for example since individuals who test frequently are likelier to be identified soon after infection [35], and individuals who are already diagnosed may be less likely to attend VCT. However, differences in selection criteria for the two groups make comparison of testing history difficult.

Rwanda's national ART guidelines [36] recommend initiating antiretroviral treatment for all individuals with CD4<350 cells/µl (regardless of AIDS symptoms), as well as for those in the most advanced clinical disease stage (regardless of CD4 count). Based on these guidelines, 33% of newly diagnosed FSW (55% of whom had never been HIV tested), and 36% of newly diagnosed VCT clients (44% of whom had never been tested before) were already eligible for treatment because of CD4<350 cells/µl. Furthermore, the substantial proportion of women (21% FSW, 15% VCT clients) with CD4 350–499 cells/µl who reported recent AIDS symptoms suggests potential additional treatment eligibility in this group. All HIV-positive participants were referred for care and evaluation for treatment eligibility.

Pregnancy prevalence in both groups was high, and the substantial proportion of currently breastfeeding women suggests a high number of recent pregnancies. Despite the fact that HIV-infected FSW were significantly less likely than uninfected FSW to be currently breastfeeding, there was no significant difference in median number of lifetime pregnancies or proportion currently pregnant by HIV status. It is possible that this association between breastfeeding and HIV infection was confounded by unmeasured factors. Nonetheless, improved family planning services and education, including on condoms as a dual protection method, would benefit these women. While most FSW reported using male condoms (albeit inconsistently), use among VCT clients was very low, and there was worrisome inconsistency across partner types in both groups. Women in both groups were unlikely to use condoms with husbands or steady partners, and VCT clients reported very low levels of condom use with casual partners. Condom distribution and education campaigns should be scaled up for Rwandan FSW and other women in potentially risky partnerships of all types (marital, steady, or casual). Participants' use of contraceptive methods other than condoms was low, although somewhat higher than the DHS+ rate (9.6%) [5]. Our participants reported waiting for menses to return after childbirth and fear of side effects as main reasons for not using contraception, demonstrating an acute need for education regarding contraceptive safety and effectiveness (data not shown). All pregnant, HIV-infected women in both groups were referred to a PMTCT program for care.

This study has a number of strengths, including rapid accrual of an at-risk, hard-to-reach group (FSW), and documentation of HIV/STI prevalence rates, and collection of in-depth demographic and behavioral information, in two epidemiologically important but understudied groups. Study limitations are also noted. Data on risk behaviors are based on self-report, and therefore subject to bias. Recruitment methods for FSW, including eligibility criteria and lack of statistical representativeness of the sample to the FSW population in Kigali, may reduce the generalizability of HIV prevalence and other data. VCT clients are a self-selected group and comparisons with the general population (e.g., DHS sample) are therefore difficult. A small number in both study groups with recent infection prohibited a more thorough statistical explanation. Finally, while VCT clients were asked about engagement in transactional sex, interview data are not able to distinguish self-declared FSW among the VCT population.

Our population-based survey reveals a high HIV burden—both in terms of prevalent and recently acquired infection—among female sex workers and VCT clients in urban Kigali, the scale of which is masked by the relatively low general-population HIV prevalence in Rwanda. Timely and comprehensive data such as these on HIV prevalence, recent HIV infection and their correlates should benefit those responsible for maintaining and strengthening HIV/AIDS surveillance in Rwanda, as well as those planning prevention interventions among at-risk groups.
